# Comparison of orthopaedic manifestations of multiple epiphyseal dysplasia caused by MATN3 versus COMP mutations: a case control study

**DOI:** 10.1186/1471-2474-15-84

**Published:** 2014-03-15

**Authors:** Sang Gyo Seo, Hae-Ryong Song, Hyun Woo Kim, Won Joon Yoo, Jong Sup Shim, Chin Youb Chung, Moon Seok Park, Chang-Wug Oh, Changhoon Jeong, Kwang Soon Song, Ok-Hwa Kim, Sung Sup Park, In Ho Choi, Tae-Joon Cho

**Affiliations:** 1 Department of Orthopaedic Surgery, Seoul National University College of Medicine, 103 Daehak-ro Jongno-gu, Seoul 110-799, Korea; 2 Department of Orthopedic Surgery, Korea University Guro Hospital, Seoul, Korea; 3 Department of Orthopaedic Surgery, Yonsei University Severance Children’s Hospital, Seoul, Korea; 4 Department of Orthopaedic Surgery, Sungkyunkwan University Samsung Medical Center, Seoul, Korea; 5 Department of Orthopedic Surgery, Kyungpook National University Hospital, Daegu, Korea; 6 Department of Orthopaedic Surgery, The Catholic University of Korea, Bucheon St. Mary’s Hospital, Bucheon, Korea; 7 Department of Orthopedic Surgery, Keimyung University Dongsan Medical Center, Daegu, Korea; 8 Department of Radiology, Ajou University Hospital, Suwon, Korea; 9 Department of Laboratory Medicine, Seoul National University Hospital, Seoul, Korea

**Keywords:** Multiple epiphyseal dysplasia, COMP, MATN3, Clinical manifestations

## Abstract

**Background:**

Multiple epiphyseal dysplasia (MED) is a relatively common skeletal dysplasia mainly involving the epiphyses of the long bones. However, it is a genetically heterogeneous group of diseases sharing certain aspects of the radiologic phenotype. In surveys conducted in East Asia, MATN3 was the most common causative gene, followed by COMP. In this study, the authors compared clinical manifestation of MED patients caused by MATN3 and COMP gene mutations, as well as subsequent orthopaedic interventions.

**Methods:**

Fifty nine molecularly-confirmed MED patients were subjects of this study. The MATN3 gene mutation group comprised of 37 patients (9 female, 28 male). The COMP gene mutation consisted of 22 cases (15 females, 7 males). Medical records and radiographs were reviewed, and questionnaire surveys or telephone interviews were conducted.

**Results:**

At the first presentation, the mean age was 8.8 ± 2.8 years (mean ± standard deviation) in the MATN3 group, and 8.5 ± 3.5 years in the COMP group (p = 0.670). The height in the COMP group was significantly shorter than those in the MATN3 group (p < 0.001). Gait abnormality at the first visit (p = 0.041) and the lastest follow-up (p = 0.037) were statistically significant difference. Hip pain (p = 0.084), limitation of daily activity (p = 0.075) at the latest follow-up tended to be more frequent in the COMP group. Hip dysplasia was more common in the COMP group, having significantly larger acetabular angle (p = 0.037), smaller center-edge angle (p = 0.002), severe Stulberg classification (p < 0.001), and smaller femoral head coverage (p < 0.001).

**Conclusions:**

Clinical manifestations of MED caused by MATN3 were milder than manifestations of the COMP mutation group. These differences in clinical manifestation and prognosis justify molecular differentiation between the two genotypes.

## Background

Multiple epiphyseal dysplasia (MED) is a relatively common skeletal dysplasia mainly involving the epiphyses of long bones. MED clinically manifests as mild short stature, intermittent joint pain, angular or rotational deformity of the extremities, short hands and feet, and/or precocious osteoarthritis. Radiologic characteristics include delayed ossification, hypoplasia and irregularity of long bone epiphyses, and varying degrees of flattening of vertebral bodies [1]. It is considered to represent a continuous spectrum of severity [2]. MED is a genetically heterogeneous group of diseases sharing common radiologic and clinical phenotypes. Mutations of the gene encoding cartilage oligomeric matrix protein (COMP), matrilin-3 (MATN3), and alpha 1–3 chains of type IX collagen (COL9A1, COL9A2, COL9A3) are responsible for MED inherited as an autosomal dominant trait, [3] whereas mutations at the gene encoding diastrophic dysplasia sulfate transporter (DTDST or SLC26A2) are responsible for MED inherited as an autosomal recessive trait [4,5]. Some patients with clinical and radiographic phenotype of MED do not have mutations in these genes, [3,6-8] suggesting presence of causative genes other than these 6 genes.

Studies of Caucasian and East Asian populations showed ethnic differences in the distribution of causative genes for MED. In an early study on Caucasian population reported the proportions of DTDST as 14%, COMP as 10%, MATN3 as 10%, and unknown as 66% in 29 MED patients [7]. Subsequent studies reported that COMP (47% and 50%) was the most common causative gene followed by DTDST (25% and 28%), while MATN3 (17%) and COL9A1, 2, and 3 (8%) comprised only a small portion of these MED patients [3,9]. On the other hand, studies on Korean [8] and Japanese [6] populations indicated that MATN3 was the most common causative gene, comprising 54.5% and 47.4% of MED patients with known genotype, followed by COMP mutations (41.8% and 36.8%, respectively). Type IX collagen genes and DTDST represented around 10% or less.

**Figure 1:**
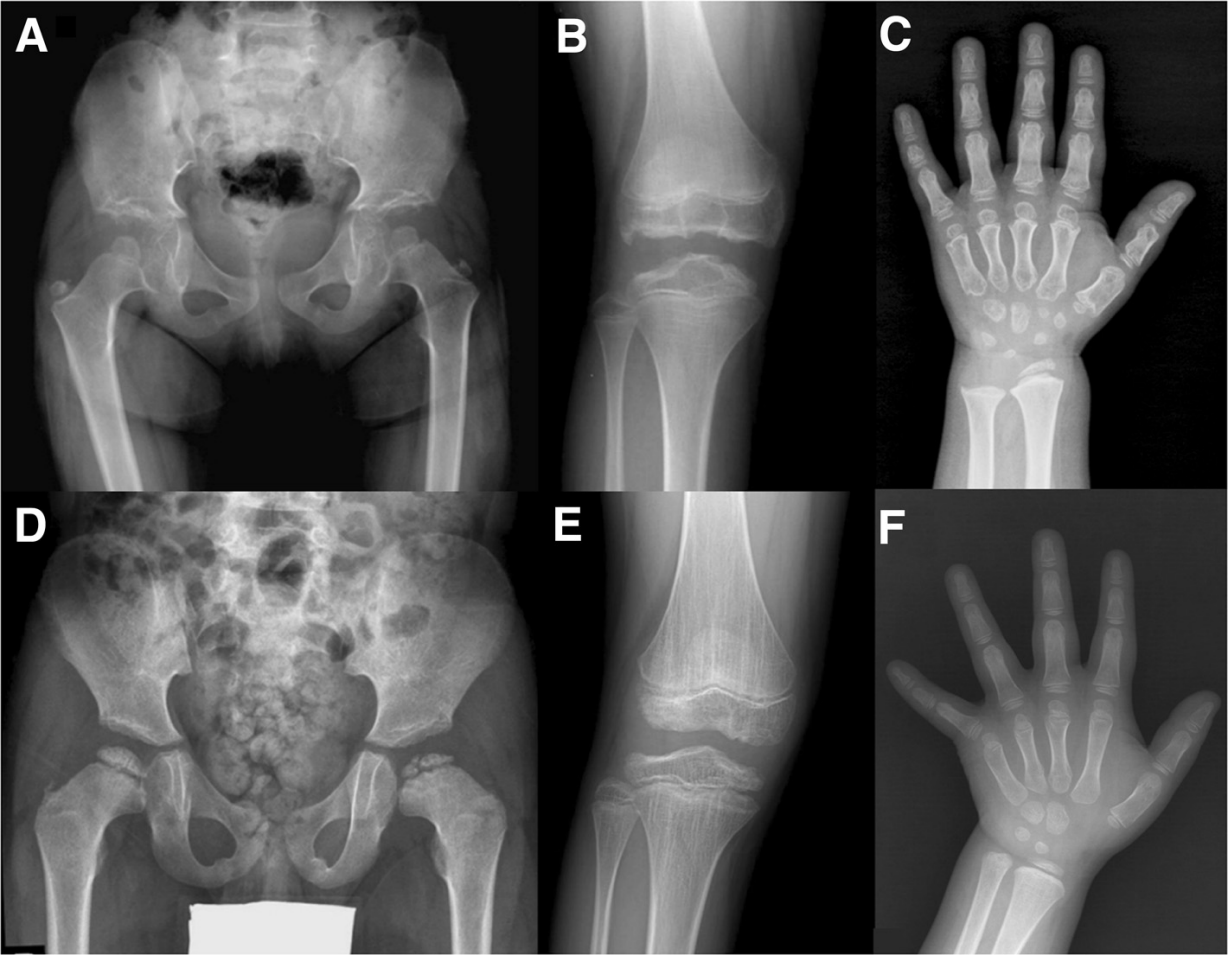
**Radiographic features of COMP-MED (A, B, C) and of MATN3-MED (D, E, F). A**: At 6 years of age, hip radiograph shows small and round femoral heads with irregular acetabular roof. **B**: At 12 years, the epiphysis at the knee joint is irregular and flattened. **C**: At 6 years, the hand radiograph shows brachymetacarpals, small and irregular carpal bones and distal radial epiphysis. **D**: At 6 years, the hip radiograph shows crescent shape femoral head with fragmentation and smooth acetabular roof. **E**: At 11 years of age, the vertical striations at the metaphysis are conspicuous along with irregular and flattened epiphysis. **F**: At age 6 years, the hand radiograph shows no brachymetacarpals or carpal bone irregularity. (This figure is partly a citation for images taken from “Revisit of Multiple Epiphyseal Dysplasia: Ethnic Difference in Genotypes and Comparison of Radiographic Features Linked to the COMP and MATN3 Genes” of the American Journal of Medical Genetics Part A. It is in the Nov 2011, Volume 155A, Issue 11:2669–2680 with permission).

Individual clinical and radiographic characteristics of MEDs caused by COMP, MATN3, type IX collagen genes or DTDST have been described [3,10-14]. Considering the incidence of each genotype of MED in East Asian populations, it is important to differentiate between the most common genotypes of MED – MATN3-MED (EDM5, MIM#607078) versus COMP-MED (EDM1, MIM#132400). Investigations on radiographic characteristics of MATN3-MED versus COMP-MED have allowed for radiographic differentiation between these two entities (Figure 1) [3,8]. However, clinical characteristics and prognosis have not been elucidated for these two genotypes of MED. The purpose of this study was to compare orthopaedic manifestations of MED caused by MATN3 versus COMP mutations and to subsequently delineate the implication of genotypes in MED.

## Methods

This retrospective study was approved by the institutional review board at Seoul National University Hospital. Fifty-nine MED patients, who were molecularly confirmed to have either MATN3 or COMP mutations, were subjects in this study.

The MATN3 group was comprised of 37 subjects (9 female, 28 male). Thirty patients were probands, and the remaining 7 subjects were affected family members of 7 probands. They harbored 7 different mutations in the MATN3 gene as previously reported [8]. The most common mutation was c.361C > T (p.R121W), present in 27 out of the 37 patients in the MATN3 group.

The COMP group was comprised of 22 subjects (15 female, 7 male). Twenty patients were probands, and the remaining 2 subjects were affected family members of 2 probands. They harbored 16 different mutations in the COMP gene as previously reported [8]. The mean follow-up period was 4.0 ± 3.9 years (mean ± standard deviation) for the MATN3 group and 6.6 ± 5.3 years for the COMP group.

Medical records were reviewed. Data at the first presentation were recorded, including age, gender, chief complaint, presence or absence of joint pain, and gait abnormalities (waddling, in-toeing, or out-toeing). The most concerning symptom at first presentation was selected as the chief complaint. Data at latest follow-up were recorded, including height, presence or absence of joint pain, and gait abnormality. Height was converted into z-score using age-matched reference data of the same ethnic group. The patients were asked as to whether the current symptom(s) interfered with their daily life.

**Figure 2:**
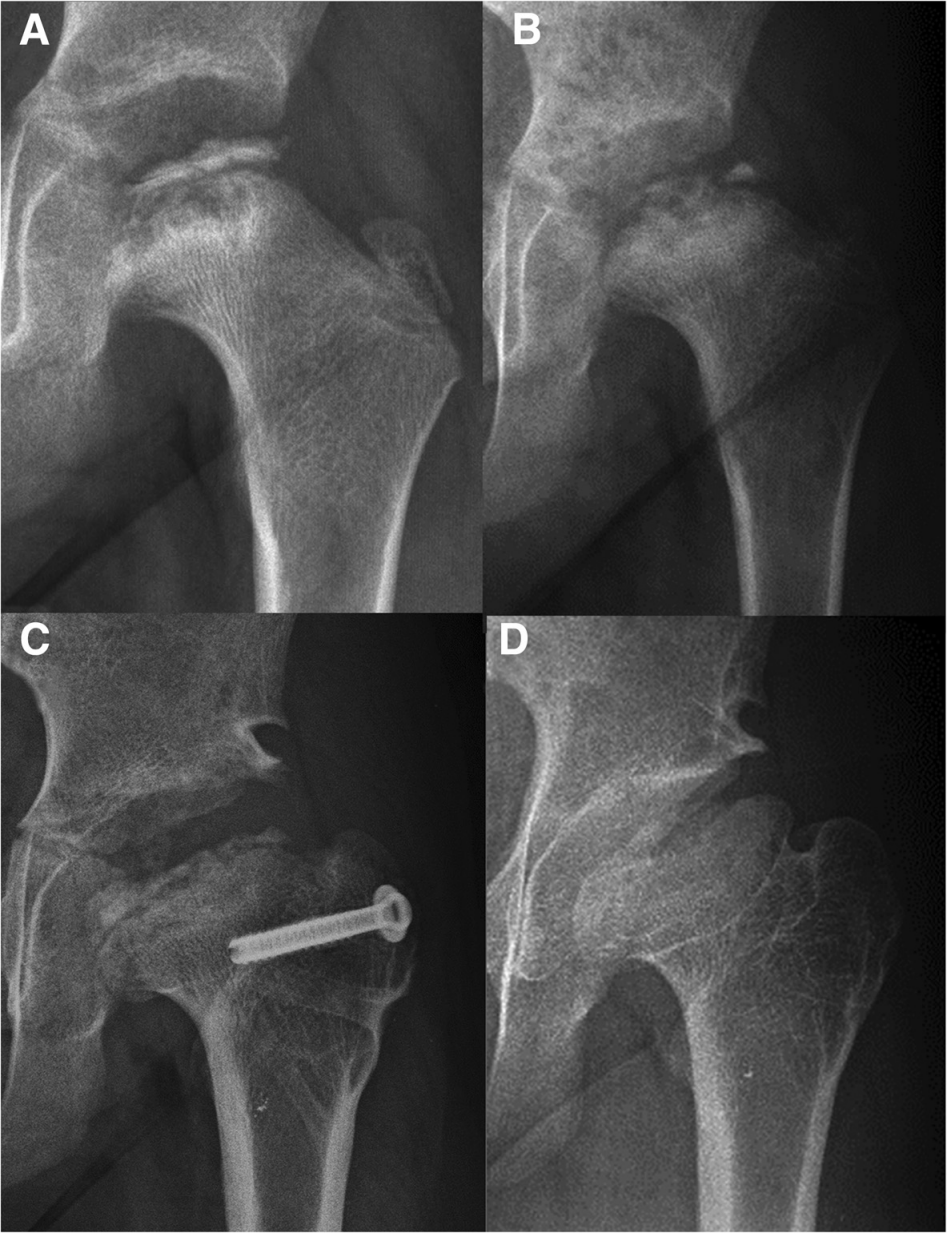
**Sequential radiographic change of AVN in an MATN3-MED patient. A**: Sclerotic femoral head and metaphyseal cyst at 9 years of age. **B**: Resorption phase at 10 years. **C**: Resossification phase at 12 years. **D**: Late reossification phase at age 15 years.

**Figure 3:**
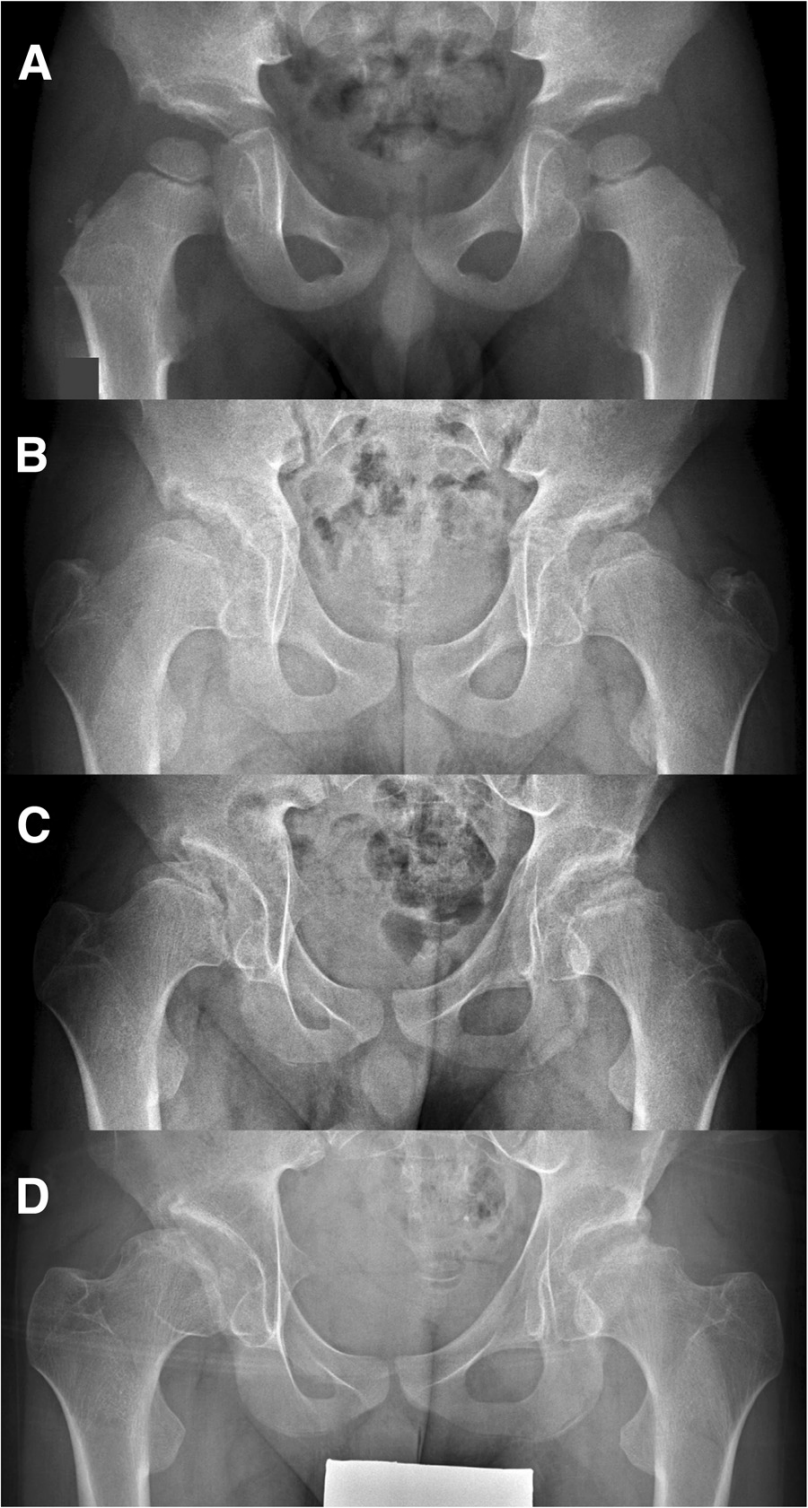
**Aberrant hip development in a COMP-MED patient. A**: At 3 years of age, the radiograph shows small and round femoral heads and irregular acetabular roof. Through the puberty and adolescence (**B**: At 11 years. **C**: At 12 years. **D**: At 13 years), the hips become dysplastic, uncovered and of flattened and irregular femoral heads.

Sequential radiographic studies of hip joints were evaluated for occurrences of avascular necrosis (AVN) of the femoral head and of aberrant development of the hip joint. AVN was defined as 1) sequential radiographic change of all or part of stages including sclerosis, subchondral crescent sign, resorption of the preexisting bone tissue, and re-ossification (Figure 2), and 2) associated painful limitation of hip joint motion during active phase. Symmetrical appearance of fragmented or aberrantly ossified femoral head without subsequent changes of avascular necrosis and/or not associated with painful limitation of hip motion was considered as aberrant development of the hip, rather than avascular necrosis (Figure 3).

In order to evaluate acetabular development and femoral head coverage, acetabular angle (AA), [15] center-edge angle (CEA), [16] Stulberg classification, [17] and femoral head coverage [18,19] were measured on the latest radiograph or on the radiograph taken just before any operation that would have changed these parameters. For statistical analysis, one hip was randomly selected on radiographs taken over 10 years of age. Twenty-seven patients in the MATN3 group were included in this analysis, whose median age was 14 years (range, 10 to 41 years), and sixteen patients in the COMP group, whose median age was 14 years (range, 15 to 46 years).

Type and purpose of the surgical intervention were categorized and recorded.

Age at the first visit of probands was compared between the two groups using student’s *t*-test. Categorical parameters were analyzed using Pearson’s Chi-square test when all the cells had frequency more than 5, or using Fisher’s exact test when any cell had frequency of 5 or less. Distribution of head deformity according to the Stulberg classification was compared using linear by linear association test. The z-score for height, AA, CEA, femoral head coverage, and number of surgical procedures were compared by the Mann–Whitney test. Variance of height and radiographic parameters were compared between the two groups using Levene’s test. A *p*-value less than 0.05 was considered as statistically significant.

## Results

The mean age at first presentation was 8.8 ± 2.8 years (mean ± SD) in the MATN3 group and 8.5 ± 3.5 years in the COMP group (p = 0.670). Chief complaints included gait abnormality, knee angular deformities, pain at the hip, knee, or ankle joints, and short stature. In the MATN3 group, pain at the knee joint was the most common chief complaint, followed by pain at the hip joint, genu valgum deformity, and gait abnormality in decreasing order of frequency. In the COMP group, gait abnormality was the most common chief complaint, followed by hip pain and knee pain. There were no statistically significant differences in proportions of chief complaints between the two groups. Of the clinical manifestations detected by interview or physical examination at the first visit, gait abnormality was significantly more common in the COMP group than in the MATN3 group (p = 0.041), but incidences of various types of pain were not significantly different between the two groups (Table 1).

**Table 1:** Clinical manifestations at the first visit

	**MATN3 group**	**COMP group**	**p-value**
Number of patients	37	22	
Age (year)	8.8 ± 2.8*	8.5 ± 3.5*	0.670***
Hip pain	11 (29.7)**	6 (27.3)**	0.844^+^
Knee pain	13 (35.1)**	10 (45.5)**	0.441^+^
Ankle pain	3 (8.1)**	4 (18.2)**	0.300^++^
Back pain	0 (0)**	0 (0)**	1.000^++^
Gait abnormality	15 (40.5)**	15 (68.2)**	0.041^+^

*Mean ± standard deviation, **number of patients (percentile).

***student's t-test, ^+^Pearson’s chi-square test, ^++^Fisher’s exact test.

At the latest follow-up, persistent gait abnormality was significantly more common in the COMP group than in the MATN3 group. Persistent hip pain and limitation of daily activity tended to be more common in the COMP group than in the MATN3 group, although it was not statistically significant. However, the prevalence of pain at joints besides the hip joint was not significantly different between the two groups (Table 2).

**Table 2:** Clinical manifestations at the latest follow-up

	**MATN3 group**	**COMP group**	**p-value**
No. of patients	37	22	
Follow-up (year)	3.7 ± 3.9*	6.6 ± 5.2*	
Hip pain	3 (8.1)**	6 (27.3)**	0.084^+^
Knee pain	7 (18.9)**	5 (22.7)**	0.748^+^
Ankle pain	2 (5.4)**	1 (4.5)**	0.887^+^
Back pain	1 (2.7)**	0 (0)**	0.445^+^
Gait abnormality	4 (10.8)**	8 (36.4)**	0.037^+^
Limitation of daily activity	4 (10.8)**	7 (31.8)**	0.075^+^

*Mean ± standard deviation, **number of patients (percentile).

^+^Fisher’s exact test.

**Figure 4:**
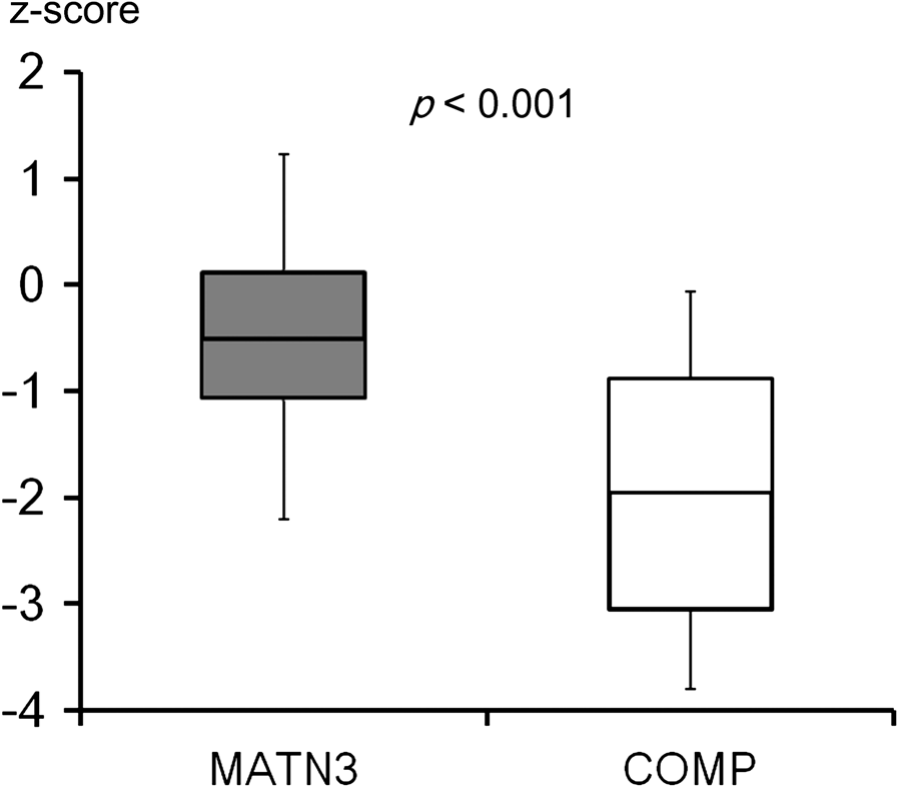
**Comparison of height between MATN3 and COMP groups.** Zero on the Y-axis denotes age and gender-matched average height.

Patients in the COMP group were significantly shorter than those in the MATN3 group (p < 0.001) (Figure 4). All the COMP-MED patients were below average in height, while twelve of 37 MATN3-MED patients were above average for their age and gender. The heights of these 12 patients range from the 50th to 89th centiles (z-value 0.02 to 1.23) of the ethnicity-specific age/gender matched normal population. The COMP group showed wider height distribution than the MATN3 group, although it was not statistically significant (p = 0.057).

**Figure 5:**
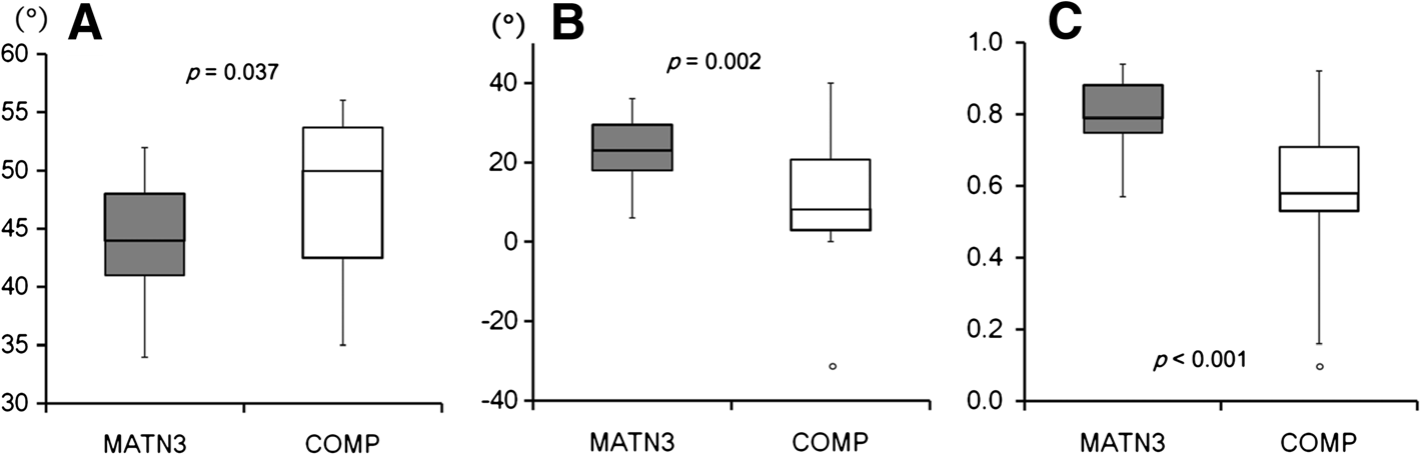
**Comparisons of radiographic parameters for hip dysplasia. A**: Acetabular angle. **B**: Center-edge angle. **C**: Femoral head coverage.

According to the criteria described above, AVN of the femoral head had occurred in 4 of 37 (10.8%) MATN3-MED patients, and 1 of 22 (4.5%) COMP-MED patients (p = 0.641). On the other hand, aberrant development of the hip was commonly observed in the COMP group, but most MATN3-MED patients developed with normal-appearing hip joints unless complicated by the AVN. Among patients older than 10 years, the COMP-MED patients exhibited more dysplastic hip joints. AA was significantly larger in the COMP group (median 50°, range 35° - 56°, standard error of the mean[SEM] 1.7) than in the MATN3 group (median 44°, range 34° - 52°, SEM 0.9) (p = 0.037), and CEA was significantly smaller in the COMP group (median 8°, range -22° to 40°, SEM 3.7) than in the MATN3 group (median 23°, range 6° to 36°, SEM 1.4) (p = 0.002) (Figure 5). Femoral head coverage was smaller in the COMP group (median 0.58, range 0.16 to 0.92, SEM 0.04) than in the MATN3 group (median 0.79, range 0.57 to 0.94, SEM 0.02) (p < 0.001) (Figure 5). The variances of CEA and femoral head coverage were significantly larger in the COMP group than in the MATN3 group (p = 0.031 and p = 0.048, respectively). And the COMP group was more diverse in AA, but it was not statistically significant (p = 0.080). According to the Stulberg classification, the severity of hip deformity was significantly worse in the COMP group than in the MATN3 group (p < 0.001) (Table 3).

**Table 3:** Stulberg classification of acetabulum and femoral head

Stulberg classification*	**MATN3 group**	**COMP group**
I	9	0
II	5	0
III	11	6
IV	2	10
V	0	0

*Linear by linear association test shows significant difference between the two groups (p < 0.001).

For statistical analysis, only one side of the hip was selected randomly on radiographs taken over 10 years of age.

A total of 76 surgical procedures were performed in 37 MATN3-MED patients, and 62 procedures in 22 COMP-MED patients. Correction of angular deformity at the knee joint was the most commonly performed procedure. Hemiepiphyseal suppression using stapling or percutaneous epiphysiodesis using transphyseal screw (PETS) were performed for growing children, while acute correction by osteotomy or gradual correction by distraction osteogenesis were reserved for the skeletally mature patients (Table 4). Hip procedures performed include shelf acetabuloplasty, proximal femoral osteotomy, Chiari osteotomy, triple osteotomy of the pelvis, and soft tissue release. The COMP-MED patients underwent significantly more hip surgeries than the MATN3-MED patients (p = 0.034) (Table 4).

**Table 4:** Summary of surgical procedures

	**MATN3 group**	**COMP group**	***p*-value**
Number of patients in the group	37	22	
Number of patients having operation	15 (40.5%)	13 (59.1%)	0.168*
Number of surgical procedures	76	62	0.117*
For angular deformity at the knee	38	27	0.542*
Hemiepiphyseal suppression	34	16	
Acute correction by osteotomy	2	6	
Ilizrov method	2	5	
For rotation deformity	0	1	0.373^+^
FDO	0	1	
Hip surgery for coverage/containment	6	15	0.034*
Shelf acetabuloplasty	2	5	
FVDO	3	3	
Chiari osteotomy	0	1	
Triple innominate osteotomy	0	1	
Soft tissue release	1	3	
Implant removal	32	19	0.776*

FDO: femoral derotation osteotomy, FVDO: femoral varization-derotation osteotomy.

*Mann-Whitney test, ^+^Fisher’s exact test.

## Discussion

This study compared clinical and radiological characteristics of MED caused by MATN3 mutations versus COMP mutations, from an orthopaedic point of view. Characteristics of various MED genotypes have been described in a small number of patients or in families [3,12-14,20-22]. However, this was the first study that statistically analyzed differences in clinicoradiographic features in substantial numbers of molecular-confirmed MATN3-MED versus COMP-MED patients.

The clinical characteristic of MATN3-MED has only been reported in studies of a single family [12,21,22] or a small number of patients [22]. In these studies, the investigators described MATN3-MED patients as having slightly short or normal stature, complaints in early childhood at knee and hip joints, and early onset osteoarthritis [3,12,22]. The current study confirmed that MATN3-MED patients can have above average height, and are significantly taller in comparison to COMP-MED patients (Figure 4). The MATN3 group demonstrated a significantly lower incidence of gait abnormality throughout the follow-up period, and patients were less likely have complaints of hip pain and limitation of daily activity at latest follow-up. Accordingly, our findings suggest that the MATN3 mutation results in relatively milder phenotypes as compared with those of COMP mutation. In contrast, COMP-MED is an allelic disease of pseudoachondroplasia (PSACH; MIM#177170), which is a more severe disease in the spectrum of COMP-pathy. Hence, it is likely that COMP-MED constitutes a spectrum from mild to severe MED cases. Our data also showed significantly more diverse CEA and femoral head coverage in the COMP group.

Orthopaedic intervention is frequently indicated in patients with MED. In this series, correction of angular deformity at the knee joint was the most common surgical procedure for MED, without significant differences between groups (Table 3). During childhood, hemiepiphyseal suppression by stapling, [23] PETS, [24] or guided growth should be effective in correction of the angular deformity around the knee joint. Angular deformity correction improves biomechanics and external appearance of the knees. However, these procedures do not guarantee prevention of early onset osteoarthritis of the knee because the cartilage matrix defect originates from mutations of either MATN3 or COMP. Due to the multicenter nature of this study, selection of surgical procedures depended largely on operator preference in this series.

Surgical intervention at the hip joints was less common than at the knee joints, but the prognosis for hip joints seems to be more guarded, especially in COMP-MED patients. The knee joint surgeries were angular deformity correction procedures with straightforward outcome. On the other hand, the hip surgeries were to improve hip joint function in AVN or hip dysplasia secondary to aberrant hip development, in order to increase the femoral head coverage or containment, however the efficacy has yet to be determined. In our series, AVN occurred in both groups. MATN3-MED patients, who showed abnormal radiographic findings at the hip joint during early childhood, usually developed normal-appearing hip joints unless complicated by AVN. On the other hand, many COMP-MED patients showed persistent aberrant hip development, and ended up with deformed and sometimes subluxated hip joints, which may keep the hip joints symptomatic, even at adolescence. This difference is reflected by the significant difference in the frequency of hip surgery between the two groups (Table 4). However, it is noteworthy that hip and knee joint pain complaints during childhood are not necessarily associated with AVN, but are more likely to be transient in nature and usually subside with conservative management.

Abnormal appearance of the femoral head in MED is sometimes confused with AVN, and genuine AVN also develops in MED patients, which should be strictly differentiated from the MED itself. Mackenzie et al. reported a series of AVN associated MED [25]. From that report, we would suggest the diagnostic criteria of AVN in MED as: 1) a new onset of clinical symptoms of limping, pain at groin, thigh or knee, limitation of hip motion, particularly abduction and internal rotation; 2) sequential radiographic changes of AVN, including sclerosis, subchondral “crescent” sign, resorption of existing bone tissue, and reossification process along with metaphyseal cyst or widening; 3) unilateral or asynchronous involvement; 4) documentation of cold spot at previously ossified areas of the femoral head on bone scintigraphy before resorption phase; or 5) MRI findings compatible with AVN. However, many MED patients show abnormal hip development which is not compatible with the above criteria for AVN. These abnormalities include irregular and steep acetabular roof, persistent fragmentation or mottling of the femoral head, flattened femoral capital epiphysis, and eventual deformation of the femoral head. We believe these do not constitute cases of AVN, but of aberrant hip development from skeletal dysplasia.

This study has several limitations. It was designed as a retrospective multicenter study, and clinical manifestations could only be categorized on an all-or-none basis, instead of more a sophisticated standardized scoring system. Most patients have not reached skeletal maturity at the latest follow-up; hence, this study represents interim follow-up results. Nevertheless, the study was able to demonstrate significant differences in clinical characteristics between MATN3-MED and COMP-MED.

Although MATN3-MED and COMP-MED are currently categorized under the same clinical diagnosis of MED, the long term prognosis of the joints, especially hip joint, should be different. Unless complicated by AVN, MATN3-MED patients are expected to develop normal appearing hips at skeletal maturity. In contrast, COMP-MED patients seem to experience more clinical symptoms in childhood, and are more likely to develop early onset osteoarthritis of the hip due to aberrant development.

## Conclusion

In comparison between MATN-3 and COMP-MED, the COMP-MED patients were shorter in height, and had more gait abnormality and hip dysplasia, while the MATN3-MED patients had a relatively milder phenotype from an orthopaedic point of view. These differences in clinical manifestation and prognosis justify distinguishing between the COMP and MATN3 molecular subtypes of MED.

### Consent

Written informed consent was obtained from the patient’s guardian/parent/next of kin for publication of this report and any accompanying images.

## Competing interests

The authors declare that they have no competing interests.

## Authors’ contributions

SGS collected and analyzed data and wrote the manuscript. HRS, HWK, JSS, CYC, MSP, CWO, CHJ, and KSS collected and analyzed data. WJY and IHC discussed the results and revised the manuscript. OHK analyzed data and gave radiologic support. SSP analyzed data and supported genetic analysis. TJC planned the study, collected data and wrote the manuscript. All authors edited the drafted version of the manuscript. All authors read and approved the final manuscript.

## Pre-publication history

The pre-publication history for this paper can be accessed here:

http://www.biomedcentral.com/1471-2474/15/84/prepub
